# Antibiotic-Loaded Hydrogel Coating to Reduce Early Postsurgical Infections in Aseptic Hip Revision Surgery: A Retrospective, Matched Case-Control Study

**DOI:** 10.3390/microorganisms8040571

**Published:** 2020-04-15

**Authors:** Daniele De Meo, Valeria Calogero, Lorenzo Are, Armando U. Cavallo, Pietro Persiani, Ciro Villani

**Affiliations:** 1 Department of Orthopaedic and Traumatology, Policlinico Umberto I Hospital - Sapienza University of Rome, Piazzale A. Moro, 3, 00185 Rome, Italy; valeria.calog@gmail.com (V.C.); lorenzo.are0@gmail.com (L.A.); ppersiani@me.com (P.P.); ciro.villani@uniroma1.it (C.V.); 2 Department of Biomedicine and Prevention, Tor Vergata University, Via Cracovia, 50, 00133 Rome, Italy; armandocavallo90@gmail.com

**Keywords:** infection, biofilm, hydrogel, local antibiotics, periprosthetic joint infection, hip revision, antibiotic prophylaxis, antibacterial coating

## Abstract

Periprosthetic joint infections (PJIs) are a cause of frequent implant failure in revision hip replacement surgery. The purpose of this study is to evaluate the onset of early postoperative infections in patients who underwent hip surgery with cementless prostheses treated with an antibiotic loaded hydrogel on their surface, in addition to systemic prophylaxis, and compare them to a control group. The secondary objective was to evaluate the onset of any local and systemic adverse effects and interference with bone ingrowth processes and functional recovery. A retrospective observational study was conducted on patients who underwent revision hip surgery by performing a 1:1 match between patients treated with an antibiotic hydrogel (ALH) and the control patients. The incidence of PJIs was assessed with a minimum of six months follow-up. Seventeen patients treated with the ALH were compared with 17 patients from the control group. No PJIs were reported in the ALH group versus the six cases encountered in the control group (*p* < 0.0001). No significant differences were reported with regard to prosthetic osseointegration and functional results, nor were there side effects in the ALH group. Despite the low sample size, the use of on-site prophylaxis with ALH has proven effective and safe in reducing the risk of PJIs in patients with a high risk for infections. Further studies are needed to validate these results in other implant-related surgeries.

## 1. Introduction

Periprosthetic joint infections (PJIs) are currently one of the most frequent causes of revision. According to Italian ministerial data [1], the total hip arthroplasty (THA) revisions in 2015 amounted to 6844 and, of these, PJIs are the third leading cause of revision surgery (11.4%). PJIs increase considerably among the causes of re-revision, up to 22.13% [2]: the failure of the surgical treatment for a prior infection is compounded by infections acquired during the revision surgery, as the latter is burdened by the increase in surgical times and invasiveness in areas with impaired vascular supply in patients who are on average older and with more comorbidities. In a 2017 review, in fact, out of 3555 patients who underwent hip prosthesis revision for septic and nonseptic causes, 8.13% of the cases were re-revisions due to “new infections” in patients who underwent revision surgery for aseptic causes [3].

In order to reduce the occurrence of infections, a series of methodologies, which allow antibiotics or substances with antibacterial activity to be placed locally, have been developed and classified by Romanò et al. into three groups based on the mechanism of action [4]. Currently, in the field of cementless prosthetic surgery, there is only one local carrier or coating device on the market that is applicable to all implantable devices: the Defensive Antibacterial Coating (DAC®—Novagenit, Mezzolombardo, Italy), a biocompatible hydrogel capable of bringing high concentrations of antibacterial drugs with the desired level of elution while at the same time acting as a physical barrier against bacterial adhesion [5,6]. From a molecular point of view, it is made up of covalently bound hyaluronic acid and polylactic acid and is designed to undergo complete hydrolytic degradation within 72 h. The rationale on the use of hyaluronic acid has been previously demonstrated in vitro by several authors. Hyaluronan coating has been demonstrated to change the hydrophobicity of an implant surface into a hydrophilic surface reducing significantly bacterial attachment [7]. Gasik et al. described how a hydrophobic surface might be a suitable substrate for bacterial colonization, while a hydrophilic surface can prevent biofilm formation [8]. In addition, some authors have described that biomatrices, such as Hyaluronan, can provide an antagonistic effect against hyaluronidase expressed by many pathogens to penetrate the physical defense of the host [9]. Bacteriostatic effect of Hyaluronan have also been demonstrated in different surgical applications [10].

The DAC^®^ hydrogel has shown synergistic activity with various antibiotics and antibiofilm agents [11]. Furthermore, it proved safe and effective in vivo testing in a rabbit model with a highly contaminated implant, both with and without systemic antibiotic prophylaxis [12].

The purpose of this study is to evaluate the onset of early postoperative infections in patients who underwent hip reoperation with cementless prostheses treated with an antibiotic loaded hydrogel on their surface, in addition to the systemic prophylaxis, and compare them to a control group of patients who received only systemic antibiotic prophylaxis. The secondary objective was to evaluate the onset of any local and systemic adverse effects and any interference with bone ingrowth processes and with the functional recovery of this type of patient.

## 2. Materials and Methods

### 2.1. Study Design and Population

A retrospective case-control observational study was performed on patients treated at a single medical centre (Umberto I University Hospital, Rome, Italy) who underwent hip surgery between January 1 2013 and December 31 2018. Informed consent regarding the collection and analysis of the data related to the surgery was obtained from all the individual participants included in the study.

The patients included suffered the failure of a prior THA or the failure of an osteosynthesis on a prior femoral neck fracture. Among the THA failures, we included patients with aseptic loosening, acute or relapsing dislocation, periprosthetic fracture, adverse reactions to metal debris, and wear or mechanical breakage of the prosthetic components. Within the prior osteosynthesis failures, we included patients with intra or extracapsular fracture and aseptic necrosis of the femoral head, pseudarthrosis, consolidation delay with concurrent cut-out or cut-off, implant failure, and posttraumatic arthrosis. In all the patients who were conversion arthroplasty candidates, the internal fixation devices were still in place and were removed during the hip replacement surgery.

The exclusion criteria were patients who underwent revision with cemented components, failure due to current or prior surgical site infection (superficial or deep), presence of neoplastic diseases with quoad vitam prognosis of less than 3 months, prior diagnosis of immunodeficiency or immunosuppressive therapy for organ transplantation, known allergy to antibiotics or hydrogel components, pregnancy, and breastfeeding.

We collected all preoperative clinical, laboratory, and radiographic data, the Charlson comorbidity index (CCI), and the PJI risk score [13].

The Harris hip score (HHS) was also recorded preoperatively in the chart of all the patients not suffering from acute traumatic causes.

The sample consisted of a total of 177 subjects. All the patients who underwent local antibiotic-prophylaxis treatment with the antibiotic hydrogel (antibiotic loaded hydrogel group, ALH) (N = 17) were compared with a 1:1 ratio to the patients selected through the propensity score matching [14] (control group).

### 2.2. Surgical Treatment and Hydrogel Preparation

The surgeries were performed by the same surgical team using the posterolateral approach; in selected cases, a direct lateral surgical approach was used.

The patients all followed the same pre, intra, and postoperative protocols for pain control, anesthesia, systemic antibiotic prophylaxis, and surgical wound care. The systemic antibiotic therapy protocol involved the administration of a cycle of 2-g Cefazoline, approximately 30 min before the surgical incision, with subsequent similar doses every 8 h for the first 24 postsurgical hours, with the exception of adjusted dosages for patients with kidney failure. An additional dose of antibiotic was administered in cases where the surgery lasted for more than 3 h. In patients with a known allergy to cephalosporins, 1-g Vancomycin was used approximately 2 h prior to surgery. Blood transfusions were performed in patients with hemoglobin values of <8 mg/dL; in cardiac patients the threshold value was 10 mg/dL.

**Figure 1:**
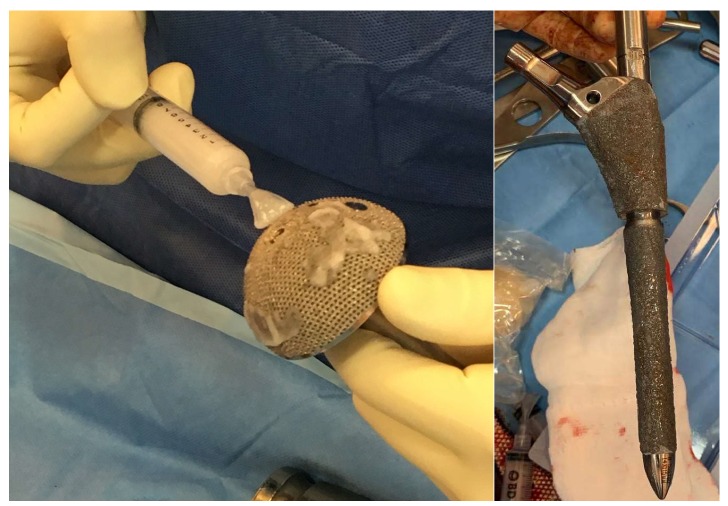
Application of Defensive Antibacterial Coating (DAC) gel on the final implant.

As for the group of patients in which the antibiotic hydrogel was used, the application of said hydrogel on the implant components took place immediately before their positioning. Gentamicin 200 mg was added to the hydrogel (5 mL). In cases where the surface could not be fully coated with the content of one vial, two vials (5 mL with added Gentamicin 200 mg and 5 mL with added Vancomycin 250 mg) were mixed together prior to use (Figure 1). Four ALH patients were treated with gentamicin + vancomycin and the rest with gentamicin only. All the patients underwent preoperative joint aspiration, anatomopathological analysis of the material taken intraoperatively, and microbiological analysis, including sonication of the explanted component for the exclusion of infection.

### 2.3. End-Points and Follow-Up

At the control visit, done for the purpose of the study and performed after a minimum of 6 months of follow-up, the patients from both groups were interviewed regarding the clinical course following the reoperation and were assessed both clinically and radiographically. The primary outcome was the onset of short-term infection, expanding the classically known concept of early infection from 3 to 6 months [15]. The diagnosis of infection was made according to the criteria proposed by the European Bone and Joint Infection Society [16]. The secondary outcome assessment tools were the mobilization of the implant at the last radiographic check-up, the postoperative functionality expressed through hip disability osteoarthritis outcome score (HOOS) and the HHS.

### 2.4. Statistical Analysis

The statistical analysis was carried out with the software RV 3.4.4 (R Core Team (2018). R: A language and environment for statistical computing. R Foundation for Statistical Computing, Wien, Austria. URL https://www.r-project.org/). For the propensity score matching analysis, a 1:1 match was performed (control group) [14], using the administration of the DAC as the dependent variable and the age, gender, body mass index (BMI), PJI risk score, CCI, length of stay, and operative time as response variables. The match was performed by using the optimal matching method, where the absolute average distance between all matched pairs is minimized.

The continuous variables were analyzed with the t-test, while the Fisher exact-test was used, where applicable, for the categorical variables. A 95% confidence interval and a statistically significant *p*-value < 0.05 were considered for all these results.

## 3. Results

Table 1 compares the data used for the selection of the 17 patients in the control group and those in the ALH group.

The average age of the patients was 74.9 ± 11.5 years and 75.9 ± 9.6, respectively. Of the 17 patients, 11 were female in the ALH group and 10 in the control group. The CCI values were 4.4 ± 1.7 and 4.4 ± 2.2, respectively. The PJI Risk Score was 25.2% ± 16.1% in the ALH group and 22.6% ± 19.8% in the control group. The average operative time was 168.2 ± 56.9 min and 166.8 ± 42.4 min, respectively.

There were no statistically significant differences between the two groups regarding parameters not included in the propensity score, such as diagnosis, time elapsed from failure to diagnosis, and the type of intervention performed (Table 2 and Table 3).

In the ALH group, the transfused units were 1.6 ± 2 on average, while in the control group they were 3.1 ± 4.2 (*p* = 0.1719). The length of hospital stay was 9.2 ± 6.4 days and 12.8 ± 19.9 days, respectively (*p* = 0.4920). One case of hematoma was encountered in the control group and was evacuated surgically. In the ALH group, two patients had a prolonged secretion of the surgical wound, with no signs of infection, which resolved itself independently. The follow-up, of at least 6 months in both groups, was 12.4 ± 5.7 months in the ALH group and 34.3 ± 21.3 months in the control group (*p* = 0.0003).

No early infections were found in the ALH group; six early infections were found in the control group (*p* = 0.0001). The total complications found were 3 in the ALH group and 11 in the control group (*p* = 0.0134). Of the five deceased, one belonged to the ALH group and four to the control group (*p* = 0.3353); in the first case, the cause of the reported death was sequelae of prolonged immobilisation in bed. Of the four deaths in the control group, three were due to periprosthetic infection complications and one to myocardial infarction (Table 4).

**Figure 2:**
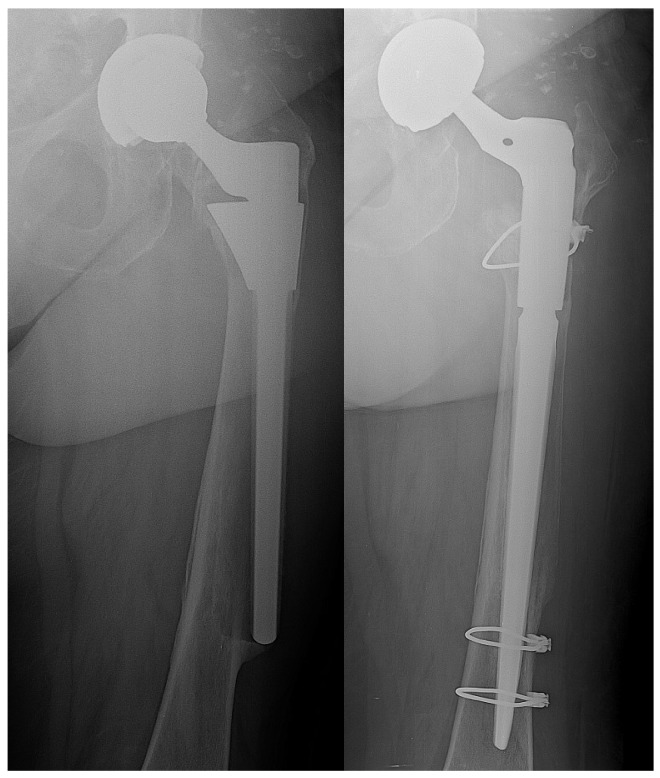
Aseptic loosening of femoral stem—stem and mobile components revision with hydrogel antibacterial coating. On the left side preoperative X-ray; on the right side five months postoperative follow-up.

Regarding the functional outcome, there were no statistically significant differences in terms of Harris hip score and HOOS at the last follow-up (*p* = 0.0687 and *p* = 0.0988, respectively) (Table 5). There were no early loosening or problems of bone ingrowth of the implant in either group (Figure 2).

All the patients who developed an infection, except for one case, were initially treated with a debridement, antibiotic and implant retention (DAIR) surgery. Three patients, two of which with failed DAIR treatment, underwent a two-stage revision (two stage exchange, TSE): two patients recovered, while the other died of sepsis (Table 6).

## 4. Discussion

The results that emerge from this study support the literature regarding the prophylactic use of ALH in cementless hip revisions. In particular, there is a statistically significant difference in the incidence of infection between the two groups analyzed.

Currently, there is no evidence on the clinical efficacy of these devices without systemic antibiotic therapy [17]. Antibiotic coatings have the advantage of ensuring high concentrations of antibiotics locally, optimizing the pharmacodynamics of these drugs [18,19] and overcoming the issues of pharmacokinetics in the bone microenvironment [20].

A lot of research has been carried out in recent years with the aim of developing and effectively applying local antibiotic release strategies. In cases where, for reasons of surgical technique or necessity, it is preferable to opt for a press-fit prosthesis, we cannot use cement as a tool for local antibiotic prophylaxis. The need to have a valid local antibiotic-prophylaxis in cementless prostheses has stimulated the research towards the development of alternative tools, such as ALH. Studies on the local application of hydrogel in prosthetic surgery seem to support the use of this type of coating [21,22,23,24].

More specifically, there is no study that focuses on aseptic hip revision. Romanò et al. analyzed the effectiveness of ALH in a prospective randomized multicenter study conducted on a total of 373 patients who underwent a first replacement or a revision surgery with cementless and/or hybrid implants (hip or knee), where they encountered a reduction in the incidence of infection in the treated group (6% vs. 0.6%; *p* = 0.003). In particular, out of 52 revision prostheses treated without the hydrogel, they encountered 4 infections (13.4%), while the outcome in the treatment group subjects was 0 out of 54. However, it should be noted that, 48 (92.3%) patients in the control group and 51 (94.4%) in the treatment group were patients who underwent the second surgery of a TSE for periprosthetic infection [21].

Other studies have shown the effectiveness of ALH against relapses or reinfections by applying the product during the reimplantation with an ongoing PJI [22,23]. Finally, a multicenter randomized clinical trial demonstrated its preventive efficacy by applying the ALH on the internal fixation implants used in the treatment of closed fractures [24]. When compared to the control population, there were no increased side effects reported in any of the studies done on hydrogel, including this one.

In cemented revisions, the use of antibiotic loaded bone cement is an on-site preventive strategy that is widely applied and supported by evidence in literature [25,26]; it can, therefore, be considered as the gold standard for reducing re-revision rates. In this type of surgery, the use of a combination of two or more antibiotics is recommended: gentamicin or tobramycin have always been used for the high elution rate characteristic of the aminoglycosides [27]; these are combined with vancomycin and/or clindamycin. The use of the combined therapy has a dual purpose: to achieve a synergistic action and reduce the growth of gentamicin resistant bacterial populations and increase the elution degree of the antibiotics themselves, obtaining higher concentrations locally [28,29]. Based on this evidence, the authors have varied the choice of antibiotic to be added to the hydrogel.

Numerous studies support the use of other classes of on-site infection control devices that are applicable on cementless prostheses, which act in a different manner compared the local release antibiotics. The use of silver-coated devices in prosthetic tumor surgery is ever-growing, with encouraging results [30]. A more recent method, which is showing promising results, is the use of iodine coated implants in neoplastic patients with severe deformities [31].

A study by Kallala et al. analyzed the effectiveness of antibiotic loaded calcium sulphate beads in 755 patients who underwent hip or knee revision surgery for septic and aseptic causes: failures for septic causes were found in 4.5% of cases [32]. Even in this case, however, it was not possible to evaluate the prophylactic efficacy alone, since many of the patients treated were already suffering from PJIs. There is also great variability regarding the safety profile of these devices in terms of the onset of hypercalcemia, heterotopic ossifications and prolonged secretion from the surgical wound [33,34,35]. This variability of side effects specific to the product probably depends on the versatility of preparation in terms of sphere size, quantity of product, and positioning of said product (on the fascial, subcutaneous, and intra-articular plane); meticulous planning would therefore be necessary. In addition, these beads are antibiotic carriers and not real coatings as they do not adhere to the surface of the implant in order to protect it.

Currently, one of the issues limiting the diffusion of coating prevention strategies is the lack of large randomized clinical trials that prove their effectiveness in the orthopedic field [17]. Additionally, numerous criticisms have been raised regarding the use of these devices, due to their high costs in the context of prevention. In a cost–benefit analysis, some Italian authors have simulated the cost-saving that would occur if on-site prophylaxis strategies were routinely used [36]: in their simulation, the authors highlighted that, currently, the routine use of silver-coated devices in prosthetic tumor surgery is not cost effective, while the routine use of the hydrogel or antibiotic loaded bone cement would be economically beneficial.

The present study has various limitations, being retrospective and not having made use of randomization for the division of the two groups of treated subjects. Additionally, this study included patients who underwent conversion prosthesis due to the failure of previous synthesis, which are currently considered on par with primary prostheses. This study however, must be taken into account in light of recent literature evidence that considers said surgical interventions, in terms of duration of the surgical act and of intra and postoperative complications, on par with revision prostheses [37]. Another limit to consider is the duration of the follow-up that, though done for a minimum of six months, turns out to be a variable with a statistically significant difference between the ALH group and the control group. Although the authors have considered this follow-up to be sufficiently lengthy enough to detect the onset of a postoperative infection, it may not be sufficiently lengthy enough to exclude any low-grade infections. However, we must point out that the PJI encountered in the control group were all considered early infections and occurred post surgically within six months. Randomized clinical trials with lengthier follow-ups and greater sample size are therefore necessary in order to confirm the data that emerged from the study.

## 5. Conclusions

This currently represents one of the few studies that analyze the effectiveness of ALH on a selected sample of patients that does not include first implant prostheses nor prostheses that failed for septic causes, also prior treated; in particular, the exclusion of the latter patient population made it possible to analyze the effectiveness of the local antibiotic-prophylaxis without bias related to the presence of an ongoing infection or bacterial contamination. Considering the aforementioned premises, this work, to date, is the only one that evaluates the prophylactic effectiveness of this product in aseptic hip revisions.

## Figures and Tables

**Table 1:** Parameters used for the propensity score matching.

Demographic Data	ALH	Controls	*p* Value
Age	74.9 ± 11.5	75.9 ± 9.6	0.7973
Sex (F)	11	10	1
BMI	27.1 ± 5.1	24.9 ± 4.9	0.1801
CCI	4.4 ± 1.7	4.4 ± 2.2	1
PJI risk score	25.2 ± 16.1	22.6 ± 19.8	0.6795
Operative time (min)	168.2 ± 56.9	166.8 ± 42.4	0.9348

**Table 2:** Diagnosis and time-to-failure.

	ALH	Controls	*p* Value
**Periprosthetic Fracture**	5	7	0.7207
**Aseptic Loosening**	4	4	1
**Dislocation**	3	1	0.6012
**Wear**	1	2	1
**ARMD ^1^**	0	1	1
**Fixation Failure**	4	2	0.6562
**Time-To-Failure (month)**	103.7 ± 108.3	112.1 ± 96.8	0.8150

^1^ Adverse Reaction to Metal Debris.

**Table 3:** Type of surgical intervention.

Surgical Intervention	ALH	Controls	*p* Value
**Stem or shell revision**	5	9	0.2960
**Stem and shell revision**	1	0	1
**Mobile components revision**	1	2	1
**Osteosynthesis (only)**	5	4	1
**Conversion THA**	4	2	0.6562
**+ Bone grafting**	3	1	0.6012

**Table 4:** Complications and deaths.

Outcome	ALH	Controls	*p* Value
**Total complications**	3	11	0.0134
**Infection**	0	6	0.0001
**Dislocation**	0	1	1
**Prolonged wound discharge**	2	1	1
**Nerve deficit**	0	1	1
**Systemics**	1	2	1
**Deceased**	1	4	0.3353

**Table 5:** Pre and postoperative functional outcomes.

Functional Outcome	ALH	Controls	*p* Value
**HHS pre**	34.1 ± 29.8	38.4 ± 13.7	0.7291
**HHS post**	72.9 ± 12.9	57.6 ± 23.8	0.0687
**HOOS post**	73.6 ± 15.8	57.9 ± 26.3	0.0988

**Table 6:** Descriptive table of patients who developed postoperative periprosthetic joint infections (PJI).

Pz	Age	Sex	Diagnosis	Treatment	Prophylaxis	Time to Infection	Type of Infection	Infection Treatment	Complication	Outcome
**1**	66	M	Aseptic Loosening	Revision	Cefazolin 2000 mg	2 d	*MRSA*	DAIR then TSE	Cerclage Breakage	Cured
**2**	79	F	ARMD	Revision	Vancomycin 1000 mg	35 d	*Klebsiella P.*	DAIR then TSE	Sepsis, MOF	Death
**3**	86	F	PPFX	ORIF	Cefazolin 2000 mg	5 m	*E. Faecalis*, *MRSA*	DAIR	Sepsis, MOF	Death
**4**	86	M	PPFX	ORIF	Cefazolin 2000 mg	14 d	*E. Coli*, *MSSA*	DAIR	NONE	Cured
**5**	86	M	Wear	Revision	Cefazolin 2000 mg	6 m	Culture Negative	DAIR	P.E.	Death
**6**	72	M	Fixation Failure	Conversion	Cefazolin 2000 mg	4 m	*MSSA*	TSE	NONE	Cured

ARMD: adverse reaction to metal debris; PPFX: periprosthetic fracture; ORIF: open reduction internal fixation; *MRSA: methicillin resistant staphylococcus aureus*; *MSSA*: *methicillin sensitive staphylococcus aureus*; DAIR: debridement, antibiotic, implant retention; TSE: two-stage exchange; MOF: multi organ failure; and PE: pulmonary embolisms.
